# Identification of peste des petits ruminants virus along with co-infecting diseases of goats in Bangladesh

**DOI:** 10.5455/javar.2022.i615

**Published:** 2022-09-30

**Authors:** Sajeda Sultana, Munmun Pervin, Nazneen Sultana, Mahbubul Pratik Siddique, Md. Rafiqul Islam, Mohammad Abu Hadi Noor Ali Khan

**Affiliations:** 1 Department of Pathology, Faculty of Animal Science and Veterinary Medicine, Sher e Bangla Agricultural University, Dhaka, Bangladesh; 2 Department of Pathology, Faculty of Veterinary Science, Bangladesh Agricultural University, Mymensingh, Bangladesh; 3 Department of Microbiology and Hygiene, Faculty of Veterinary Science, Bangladesh Agricultural University, Mymensingh, Bangladesh; 4 Animal Health Division, Bangladesh Agricultural Research Council, Dhaka, Bangladesh

**Keywords:** Co-infection, goat, PCR, RT-PCR, PPRV

## Abstract

**Objective:**

Peste des petits ruminants (PPR) virus is the main infectious cause of goat mortality in Bangladesh, and co-infection may make diseases more severe. This study aimed to detect PPR and co-infecting diseases in goats.

**Materials and Methods:**

One hundred goats suspected to be infected with the PPR virus were collected from various areas of Mymensingh district, Bangladesh. A systemic post-mortem examination was carried out on PPR-suspected goats. Lungs, spleen, and lymph nodes (pre-scapular) were used for ribonucleic acid extraction, whereas lungs and mesenteric lymph nodes were used for deoxyribonucleic acid extraction. Seven-pair primer sets were used for molecular detection of pathogens specific for PPR, goat pox, contagious ecthyma (Orf), foot and mouth disease (FMD) virus, *Klebsiella* sp., and *Mycobacterium* sp. Reverse transcriptase-polymerase chain reaction (RT-PCR) or polymerase chain reaction (PCR) were used to find the exact cause.

**Results:**

Out of 100 PPR-suspected goats examined, 55 goats were confirmed as PPR-detected by RT-PCR. Among the 55 PPR-positive goats, 2 were co-infected with goat pox, 2 with tuberculosis, 10 with *Klebsiella* sp. infection, and 6 with FMD as detected by PCR and RT-PCR. Moreover, 12 goats were co-infected with PPRV and fascioliasis.

**Conclusion:**

About 58% of PPR virus-infected goats were co-infected with other organisms. There is a need to design technology to detect the state of co-infectivity at its early onset and future preventive and therapeutic strategies for co-infecting diseases. This is the first study in Bangladesh to describe co-infecting diseases of goats along with PPR.

## Introduction

Peste des petits ruminants virus (PPRV) causes PPR disease in small ruminants [1,2], also called the goat plague, which is economically concerning and devastating for goats. In Bangladesh, PPR outbreaks in sheep and goats have been documented since 1993 [3]. Clinically, PPR is a transboundary, acute, highly infectious, immunosuppressive viral disease that infects sheep and goats and is reportable to the OIE [4]. Fever, oculonasal discharges, necrotizing and erosive stomatitis, severe enteritis, and pneumonia are its distinguishing features [5]. However, PPRV is an ribonucleic acid (RNA) virus with a single strand and no segments. It belongs to the genus *Morbillivirus* and the family Paramyxoviridae. It has antigenic links with rinderpest, canine distemper, and human measles [6].

The morbidity rate in goats due to PPR may reach up to 100% in a sensitive population, and the mortality rate ranges from 20%–100% [7]. PPRV infection frequently leads to severe immunosuppression, which allows for the development of opportunistic secondary infections, resulting in higher morbidity and mortality rates [8]. Concurrent infection increases the severity of diseases [9]. Multiple pathogens working together (bacteria, viruses, parasites, fungi, etc.), host defense, stress, and environmental factors cause pneumonia [10].

Among bacterial pathogens,* Klebsiella* sp. was isolated from interstitial pneumonia and bronchopneumonia [11]. The prevalence of *Klebsiella* sp. was 5% in Black Bengal goats of Bangladesh [11].

Another bacterial disease, tuberculosis (TB), negatively affects the economy and public health. TB in goats and sheep is caused by members of the *Mycobacterium tuberculosis* complex, predominantly by *Mycobacterium bovis* and *Mycobacterium caprae* [12–16]; in very few cases, *M. tuberculosis* (MTB) [16–17].

Regarding parasitic diseases, the prevalence of fascioliasis in Bangladesh has been reported to vary from 10% to 32% in live animals [18] and in slaughtered animals ranging from 3.8% to 22% in goats [19]. Both PPR and fascioliasis cause severe diarrhea in goats. The PPR instances were more remarkable in non-vaccinated goats (44.23 %) than in vaccinated goats (28.57%), and the total prevalence of fascioliasis was 28.95%, with a higher prevalence (40%) in adult goats older than 1.5 years [20].

Diseases like *capripox*, contagious ecthyma (Orf), bluetongue, foot and mouth disease (FMD), and contagious caprine pleuropneumonia are close to the clinical signs of PPR [21–24] and endemic in the caprine population.

Among the viral diseases, goat pox and Orf are important diseases of goats and endemic in Bangladesh [25–26]. Conjunctivitis, respiratory distress, pyrexia, lymphadenopathy, and generalized cutaneous and internal pox lesions are the predominant clinical signs of goat pox [27–29].

In the same way, lesions in the mouth, tongue, lips, and teats are important for diagnosing Orf [30,31]. FMD is a viral disease that spreads quickly among cloven-hoofed animals, showing symptoms of fever, vesicle formation on the lips, tongue, interdigital tissue, and teats [32,33]. FMD in cattle and buffalo is endemic in Bangladesh and typically causes epidemic tremors yearly [34]. Goats are rarely vaccinated for FMD due to mild infection and the absence of severe symptoms associated with mortality. The role of goats in FMD is mainly transmission, acting as a short-term reservoir and maintaining this virus in the host [35].

Natural mixed infections due to the goat-pox virus (GTPV) with PPRV have been reported in Congo and India [9,36]. Several bacterial diseases have also been reported due to the PPR virus in Tanzania [37]. It is assumed that the high mortality rate in goats may be caused by the presence of PPR and other co-infections [36–38]. To identify the responsible pathogen(s), a differential diagnosis using appropriate laboratory tests is required [39]. In Bangladesh, very little research was carried out describing co-infecting diseases of goats.

Based on the above information, the goal of this study was to use reverse transcriptase-polymerase chain reaction (RT-PCR) and PCR techniques to find PPR viral infections in goats and concurrent infections.

## Materials and Methods

### Ethical approval

The research project with the number BAURES/ESRC/VET/07-1 was approved by the ethical standards committee of the Bangladesh Agricultural University Research System (BAURES) on January 20, 2019.

### Examination of suspected goats and collection of samples

A total of 100 goats suspected to be infected with PPR were examined. The male and female goats aged between 2 months and 3 years, naturally infected, and clinically exhibiting purulent nasal discharges, diarrhea, and dehydration were investigated during the winter season between January 2019 and March 2021. 50 PPR-suspected dead goats and their disease history were collected from goat owners, and 50 PPR-suspected live goats were collected from different areas of Mymensingh. The PPR-affected live goats were slaughtered. At post-mortem examination, the portions of the lungs, spleen, and lymph nodes (pre-scapular and mesenteric lymph nodes) were collected. These samples were collected aseptically in a sterile falcon tube, snap-frozen, and stored in a −20°C freezer until they could be used for RT-PCR and PCR to find specific organisms.

### RNA/*deoxyribonucleic acid* (DNA) extraction

The RNA was harvested from pre-scapular lymph nodes, spleen, and lungs to identify the PPR virus. RNA was harvested from PPR-positive mesenteric lymph nodes to identify the FMD virus. DNA was harvested from PPR-positive lungs and mesenteric lymph nodes to identify pox virus, orf virus,* Mycobacterium* sp., and *Klebsiella* sp.

About 20–25 mg of tissues was crushed in the sterile pestle and mortar with liquid nitrogen. Following the manufacturer’s protocols, RNA and DNA were isolated from these tissues using a commercially available RNA isolation kit (SV Total RNA Isolation System, Promega, USA) and a DNA isolation kit (Wizard Genomic purification kit, Promega, USA). Using a Nanodrop^TM ^spectrophotometer, the cleanliness and concentration of the isolated DNA and RNA were determined at 260 nm and 280 nm (IAEA, Scibersdoff, Vienna). RNA concentration of 2.0 and DNA concentration of 1.8 was assessed as pure and used for the RT-PCR and PCR to detect a specific gene of the microbes.

### Amplification of nucleic acid by RT-PCR and PCR techniques

The designed and published primer sequences (Table 1) were obtained from AIT Biotech, Singapore. The reaction volume for both the RT-PCR and the PCR was 50 μl. The RT-PCR protocol used the Verso 1-Step RT-PCR Reddy Mix kit (Thermo Scientific MA, USA) and the PCR protocol used the GoTaq G2 Green master mix kit (Promega, Madison, WI, ). The previous repository samples were used as the positive control. The reaction mixture added nuclease-free water instead of template RNA or DNA as a negative control. The reaction was conducted in an oil-free thermal cycler (ProFlex gradient PCR, USA). The RT-PCR amplification of the targeted nucleoprotein (*N*) gene of the PPR virus and the leader proteinase (*Lpro*) gene of the FMD virus began with the reverse transcription at 50°C for 15 min. Initial denaturation was performed at 95°C for 2 min, and after that, 40 cycles of amplification reaction comprised denaturation at 95°C for 20 sec, annealing at 55°C for 30 sec (*N* gene) and 55°C for 60 sec (*Lpro* gene), elongation at 72°C for 1 min, and final elongation at 72°C for 5 min. The 35 cycles (Poxvirus), 32 cycles (Orf virus), 40 cycles (MTB), 32 cycles (*M. bovis*), and 35 cycles (*Klebsiella sp.*) of PCR amplifications were carried out using initial denaturation at 95°C for 2 min and then, denaturation at 95°C for 30 sec, annealing at 50°C for 45 sec (GTPV), 58°C for 30 sec (Orf virus), 62°C for 2 min (16srRNA gene, MTB complex), 56°C for 1 min (*MPB83* gene, *M. bovis*), and 62°C for 1 min (*gyr-B-2* virulent gene, *Klebsiella* sp.) and extension at 72°C for 1 min. Final elongation was carried out at 72°C for 5 min. The cDNAs acquired through RT-PCR and PCR were electrophoresed (WSE-1710Submerge-Mini2322100, China) in a 1.5% agarose gel containing ethidium bromide (0.5 μg/ml). A transilluminator was used to take the pictures (Alpha Imager, USA). The control lane of agarose gels was loaded with a 100 bp DNA ladder (TrackIt, Invitrogen, New York, NY, ) to measure the size of cDNA. Positive cDNA samples were sequenced from Macrogen, Korea, and identified by an online Basic Local Alignment Search Tool (BLAST) search to ensure the specificity of the pathogens.

**Table 1.** Oligonucleotide primers for RT-PCR and PCR detection of specific microbial genes.

Target genes/organisms	Primers name	Sequences (5´−3´)	Amplicon size/organism name	Genbank accession no. or references
Nucleoprotein/PPRV	PPRV NF	gctctgtgattgcggctgagc	402 bp/PPR	[40]
	PPRV NR	cctggtcctccagaatcttggcc		
Envelop protein*/*Pox	PoxF1	gcgaaatttcagatgtagttc	287 bp/goat pox	Designed KY389314.1
	PoxR1	ccgcatcagcatacgatttcc		
Major envelope protein/Orf	OrfF1	cagcttctgctgcaacctgag	587 bp/Orf (Contagious ecthyma)	Designed KX129982.1
	OrfR1	gcttgatcaccggcaccatcg		
Leader proteinase/ Lpro, FMD	FMD LproF	cttctacgcctgaataagcg	430 bp/FMD	[34]
	FMD LproR	gatgatacttcccgtgttgc		
16S rRNA/TB	TB 1–F3	gaacaatccggagttgacaa	372 bp/ MTB	[41]
	TB 1–R3	agcacgctgtcaatcatgta		
MPB83/*M. bovis*	MPB83F	cagggatccaccatgttcttagcgggttg	600 bp/ *M. bovis*	[42]
	MPB83R	tggcgaattcttactgtgccggggg		
gyr-B-2/*Klebsiella*	Gyr-B-2F	tccggcggtctgcacggcgt	411 bp/ *Klebsiella* sp.	[43]
	Gyr-B-2R	ttgtccgggttgtactcgc		

## Results

### Clinico-pathological findings

The clinical history and signs observed while examining 100 goats suggested PPR viral infectivity. PPR-suspected goats showed pyrexia (104–105 °F), anorexia, severe dehydration, nasal discharges, emaciation with sunken eyes, and tachycardia. Infected goats showed watery feces adhered to the anal region. Subnormal temperatures are also noticed in terminal cases. Erosion around the mouth and tongue was also observed in some goats. The goats showed conjunctivitis, purulent nasal discharge, dyspnea, and coughing in the advanced stages of many suspected PPR cases. The skin and footpads of goats appeared healthy.

A systemic post-mortem examination was carried out on the goats. The predominant lesions observed were zebra striping lesions in the ileum of 70% of the infected goats. Congestion and hemorrhages were seen in the trachea, spleen, liver, and kidneys. Swollen, edematous, and congested pre-scapular and mesenteric lymph nodes were seen in 73% of cases. The lungs were severely congested in most cases, with foamy exudations in the cut surfaces consolidated with red or gray hepatization in the pneumonic lungs. In typical cases, a fibrinous cast was observed in the bronchus and bronchioles. The liver showed focal necrosis and massive hemorrhage in 50% of PPRV-infected cases. Thirteen 13 PPR-suspected goat livers showed adult *Fasciola* sp. in the gall bladder, and 12 dead goat livers showed pipe-stem liver. Three PPR-suspected female goats aged between2 and 3 years showed caseous nodules in the lungs and mesenteric lymph nodes. PPR was suspected in 20 goats that showed small nodules in the lungs with pleural adhesions.

### Molecular detection of PPRV

**Figure 1.**
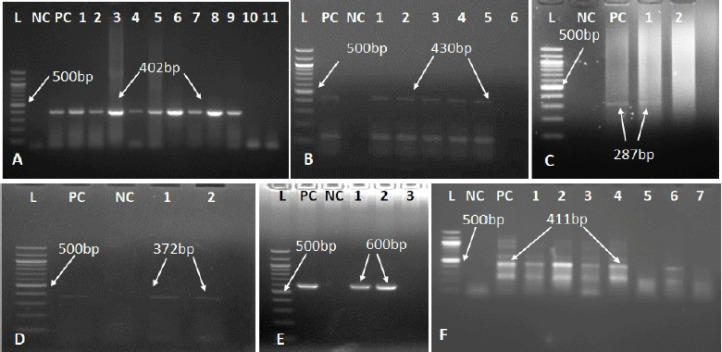
Identification of PPR virus and other co-infected organisms. (A) RT-PCR amplification of N gene of PPRV. Positive amplicons of 402 bp for PPR Virus. (B) RT-PCR amplification of *L* pro gene of FMD. Positive amplicons of 430 bp for FMD Virus. (C) PCR amplification of envelope gene of pox virus. Positive amplicons of 287 bp for pox virus. (D) PCR results for MTB. Positive amplicons of 372 bp for MTB. (E) PCR results for *M. bovis*. Positive amplicons of 600 bp for *M. bovis*. (F) PCR results for* Klebsiella* sp. infection. Positive amplicons of 411 bp for *Klebsiella* sp. *L* = 100 bp DNA Ladder, PC = Positive control, NC = Negative control, Field samples are indicated as 1 to 11.

The prevalence of PPRV in suspected goats was 55%. In the RT-PCR test, RNA from 55 goats yielded expected N gene-specific PPRV amplicons (402 bp) out of 100 goats observed (Fig. 1A). 32 dead goats and 23 suspected live goats were found PPRV-positive in the RT-PCR test. RNA from the pre-scapular lymph node, lungs, and spleen were tested for RT-PCR to detect PPRV. A goat with any organ positive for PPRV was considered PPR-positive. RNA from the lymph nodes and lungs of 20 goats (36%), RNA from the lungs and spleen of 16 goats (29%), RNA from lymph nodes and spleen of 12 goats (22%), and RNA from lungs, lymph nodes, and spleen of 7 goats (13%) were found to be PPRV-positive in the RT-PCR test.

### Co-infection detection

#### FMD detection

The prevalence of FMD and PPRV co-infection was 10.9%. Six goats were positive for FMD viral co-infection. RNA yields a 430 bp amplicon in positive cases in the RT-PCR test (Fig. 1B).

#### Pox detection

The prevalence of pox-virus and PPRV co-infection was 3.6%. Two goats were positive for pox viral co-infection. In positive cases, DNA yields a 287 bp amplicon in a PCR test (Fig. 1C). A pox-positive goat was defined as having DNA from either the lungs or the mesenteric lymph nodes amplified with 287 bp amplicons.

#### TB detection

The prevalence of TB and PPRV co-infection was 3.6%. Two PPRV-positive goats were found to be co-infected with TB. In a PCR test, DNA yields a 372 bp amplicon (Fig. 1D) in positive cases of MTB (MTB) and also yields 600 bp (Fig. 1E) for* M. bovis* in a PCR test. Any organ, lung, or mesenteric lymph node DNA amplified at 372 bp and 600 bp amplicons were considered TB-positive.

#### *Klebsiell*a sp. detection

The prevalence of *Klebsiella* sp. and PPRV co-infection was 18%. 10 goats were found to be positive for *Klebsiella* sp. co-infection. In positive cases, DNA yields a 411 bp amplicon in a PCR test (Fig. 1F). *Klebsiella* sp.-positive goats had DNA from any organ, lung, or mesenteric lymph node amplified with 411 bp amplicons.

#### Fascioliasis detection

The prevalence of fascioliasis and PPRV co-infection was 21.8%. On post-mortem examination, fascioliasis was observed in 25 goats. Out of 25 goats infected with* Fasciola* sp., 12 goats were found to be PPR-positive in an RT-PCR test.

**Figure 2.**
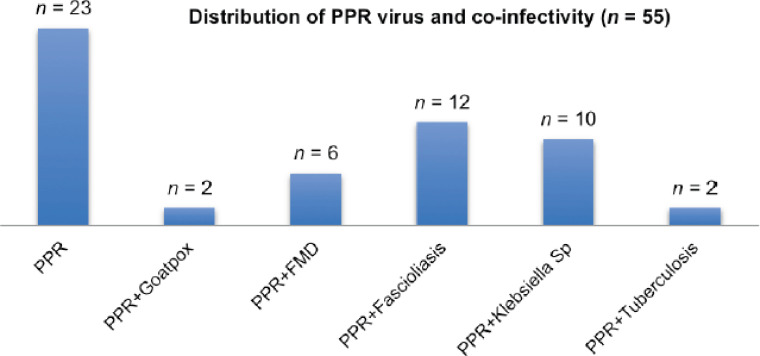
Representation of the PPR viral infectivity alone and with other co-infecting diseases. Among 55 PPR-positive samples as detected by RT-PCR, 23 goats showed PPR infection alone, 2 goats were co-infected with goatpox, 6 with FMD virus, 12 with *Fasciola* sp., 10 goats with *Klebsiella* sp., and 2 goats with tuberculosis co-infection

None of the goats was co-infected with the Orf virus. About 58% of PPR virus-infected goats were found to be co-infected with pox, FMD, fascioliasis, *Klebsiella* infection, and TB (Fig. 2). Sequenced cDNAs of PPR, FMD, and goat pox were identified by an online BLAST search and identified with 97%–98% similarity to specific classes of the viruses.

## Discussion

The only way to eradicate PPR globally is through fast outbreak investigation, notification, quick response, and immediate control measures. The incidence of actual PPRV infection and simultaneous co-infections contribute to a more severe or long-term issue [44,45]. The creation and implementation of specific diagnostic tests that can separate PPR from diseases with the same symptoms can undoubtedly help to improve information and understanding of the disease’s geographical circulation and dispersion in particular locations [46].

In this study, attention was paid to identifying co-infecting organisms in goats. A case infected with the PPR virus exhibited early clinical symptoms, including fever, depression, and ocular and nasal secretions [7]. Congestion of the gastrointestinal tract, pneumonia, engorged spleen, and swollen lymph nodes were the most notable gross lesions [7]. Lymphadenopathy is a dominant lesion in goat pox, FMD, TB, and *Klebsiella* infections [9,10,12,15,21,25,29]. This study revealed the lesions in different organs associated with lymphadenopathy at necropsy. The co-infecting diseases were identified based on post-mortem findings, PCR, and RT-PCR detection of the microbial genomes. The cadence of liver fluke infestation was detected by examining the gall bladder for the presence of adult *Fasciola* sp. and pipestem liver as a consequence of chronic fascioliasis. PPR-positive goats showed a higher rate of co-infectivity with fascioliasis. For the first time in Bangladesh, this study describes molecular detection of the PPR virus and co-infection with diseases like goat pox, FMD, TB, and *Klebsiella* sp. infection in naturally PPR-infected goats.

Since 1995, various primer sets targeting the F, M, or N proteins have been created to quickly and accurately identify PPRV by RT-PCR tests [47]. Among them, N is the principal viral protein of PPRV. Recently, many successful efforts have been made to use the N gene as a target for RT-PCR to identify PPRV [48,49]. In clinical samples, primers NF/NR (GenBank Ac.no. GQ122187.1, India, 2008) were developed to detect PPRV by selecting the N gene location at 1,130–1,151 and 1,532–1,509 base positions and producing 402 bp amplicons [40]. This study used partial N gene primers [40] to detect PPRV in goats.

Using sandwich ELISA, 60% of samples from the mixture of heart, kidney, and liver, 57.84% from the lung, 50% from the spleen, 62.5% of lymph nodes, 75% of the intestinal mucosa, 40% of nasal swabs, and 66.67% of blood samples tested positive for PPR viral antigen [50]. In this study, the prevalence of PPR viral RNA was highest in the lungs and lymph nodes (36%). During RNA extraction, RNAs may be contaminated by the organ (lungs, lymph nodes, or spleen), which was not detected in the RT-PCR test, though it were PPR-positive goats.

Small ruminants (like sheep and goats) have been demonstrated in multiple studies to be at risk for FMD and to have a role in spreading the disease by serving as temporary reservoirs [35]. In Bangladesh, vaccination rates for FMD in goats are quite low. Cattle and goats share common sheds in most cases. Viral DNA can be found in many tissues but is mostly found in lymph nodes [51]. The FMD virus is an RNA virus, and considering this point, RNA was extracted from the mesenteric lymph node. In this study, FMD was identified using RNA from the mesenteric lymph node in the RT-PCR test. Six PPR-positive goats (two dead goats and four clinically PPR-infected live goats) were positive for FMD viral co-infection. Clinical FMD lesions were not found in these cases.

The Pox virus and the Orf virus are members of the pox virus family. GTPV was experimentally infected in goats. Researchers found low viral loads in the lungs and liver; however, abundant viral DNA was found in skin crusts, nodules, and subcutaneous tissue near the injection site (Ct values: 14.6 to 22.9) [29]. For that reason, DNA was extracted from the lungs to identify the Orf virus or pox virus. This study observed no typical pox lesions in PPR-infected goats. A PCR test revealed that two PPR-positive dead goats were co-infected with the pox virus.

TB has been a common pathogen infecting humans and animals in Asian countries, including India, Pakistan, and Bangladesh, as well as in African territories. It is reported to confer a higher infection rate than in developed countries. *Mycobacterium bovis* and *M. caprae* are the two most common TB-causing bacteria in small ruminants [52], and rarely MTB [16]. Clinical symptoms of TB in small ruminants include sickness, weight loss, fluctuating temperature, intermittent hacking cough, diarrhea, and enlarged, prominent lymph nodes. The tuberculin skin test, interferon-gamma, serology, acid-fast staining, isolation of bacteria from clinical and necropsy samples, and molecular techniques like PCR and RT-PCR are only some of the diagnostic methods used to detect TB. This study used clinical signs, post-mortem examination, and PCR techniques to identify specific causes of TB. Two aged clinically emaciated goats were found to be positive for TB co-infection. The PCR protocol was used to detect TB targeting the 16SrRNA gene (372 bp) and to detect MTB Complex [41]. A single conventional PCR targeted the *MPB83* gene to identify *M. bovis* (600 bp) [42]. Cattle, sheep, and goats share grazing areas, watering places, and nighttime shelters, which increases the likelihood of interspecies transmission of *Mycobacterium* sp. germs and may result in silent infection [53,54]. In Bangladesh, cattle and goats share the common shed in most cases.

*Klebsiella* is opportunistic bacteria and have a normal habitat in healthy goats. Numerous genes contribute to *Klebsiella* species’ pathogenicity. However, the DNA gyrase subunit *B2* gene (gyr-B-2) is the primary one that has been documented by numerous researchers [55]. Clinically, nodules are found in the lungs and lymphadenopathy in most cases [12]. This study showed that the virulent gene of *Klebsiella* sp. infection could be found in the lungs and lymph nodes of 10 PPR-positive goats.

Fasciolosis in domestic ruminants has also been linked to specific demographic and seasonal risk variables [56]. Fascioliasis requires an intermediate host (*Lymnea auricularia*), and its distribution is greatly influenced by the presence of clinically or subclinically infected hosts. The *L. auricularia* snail is found throughout the year in the lowlands of tropical areas in favorable habitats [57]. Fascioliasis causes malnutrition in goats and enhances susceptibility to infection. In this study, among 32 cases of co-infection, 12 goats were co-infected with PPRV and *Fasciola* sp. (21.8%). Combined fascioliasis infection with PPR viral infectivity may cause severe diarrhea and higher mortality.

## Conclusions

The PPR virus increases morbidity and mortality rates in small ruminants. Diseases like FMD, goat pox, *Klebsiella* sp., TB, and fascioliasis were co-infected with PPRV. About 58% of PPR virus-infected goats showed co-infection. PPR-infected goats were found to be co-infected with PPRV and FMD (10.9%), PPRV and goat pox (3.6%), PPRV and TB (3.6%), PPRV and *Klebsiella* sp. (18%), and PPRV and fascioliasis (21.8%). Fascioliasis and PPR co-infection were seen at a higher rate. Co-infection may make PPR diseases more severe and cause high mortality. There is a need to design one-step molecular technology to detect the co-infectivity of similar clinical signs producing diseases at early onset and future preventive and therapeutic strategies for co-infecting diseases regarding PPR. However, this is only an early study, and more studies are required to accurately assess the prevalence of co-infectivity with PPRV in goats to minimize goat infections and maximize goat production in Bangladesh.

## List of Abbreviations

BLAST, basic local alignment search tool; DNA, deoxyribonucleic acid; FMD, foot and mouth disease; GTPV, goat pox virus; M, *Mycobacterium*; MTB, *M. tuberculosis* complex; PCR, polymerase chain reaction; PPR, peste des petits ruminants; RNA, ribonucleic acid; RT-PCR, reverse transcriptase-polymerase chain reaction; TB, tuberculosis.

## References

[1] Kashem MA, Hossain MA, Ahmed SU, Halim MA (2011). Prevalence of diseases, morbidity and mortality of black Bengal goats under different management systems in Bangladesh. Univ J Zool.

[2] Khan MAHNA (2015). A coordinated project on the surveillance of important infectious, zoonotic and emerging diseases of livestock and poultry in Bangladesh. Research review report submitted to NATP-Phase 1, PIU, BARC, Dhaka, Bangladesh.

[3] Islam MR, Shamsuddin M, Das PM, Dewan ML (2001). An outbreak of peste des petits ruminants in black Bengal goats in Mymensingh, Bangladesh.. Bang Vet.

[4] Luka PD, Erume J, Mwiine FN, Ayebazibwe C, Shamaki D (2011). Molecular characterization and phylogenetic study of peste des petits ruminants viruses from north central States of Nigeria. BMC Vet Res.

[5] Balamurugan V, Hemadri D, Gajendragad MR, Singh RK, Rahman H (2014). Diagnosis and control of peste des petits ruminants: a comprehensive review. Virus Dis.

[6] Kumar N, Maherchandani S, Kashyap SK, Singh SV, Sharma S, Chaubey KK (2014). Peste des petits ruminants virus infection of small ruminants: a comprehensive review. Viruses.

[7] Chowdhury EH, Bhuiyan AR, Rahman MM, Siddique MS, Islam MR (2014). Natural peste des petits ruminants virus infection in black Bengal goats: virological pathological and immunohistochemical investigation. BMC Vet Res.

[8] Parida S, Couacy-Hymann E, Robert AP, Mahapatra M, Medhi EH, Brownlie J (2015). Pathology of peste des petits ruminants.

[9] Malik YS, Singh D, Chandrashekar KM, Shukla S, Sharma K, Vaid N (2011). Occurrence of dual infection of peste des petits ruminants and goatpox in Indigenous goats of central India. Transbound Emerg Dis.

[10] Lacasta D, Ferrar LM, Ramos JJ, Gonzalez JM, Heras D (2008). Influence of climatic factors on the development of pneumonia in lambs. Small Rumin Res.

[11] Sukanta KS, Mohammed RC, Mahbub EE, Abu BS (2018). Bacteriological and histopathological investigation of pneumonia in black Bengal goat. Dairy Vet Sci J.

[12] Crawshaw T, Daniel R, Clifton-Hadley R, Clark J, Evans H, Rolfe S (2008). TB in goats caused by *Mycobacterium bovis*. Vet Rec.

[13] Hiko A, Agga GE (2011). First-time detection of *Mycobacterium*species from goats in Ethiopia. Trop Anim Health Prod.

[14] Mendoza MM, de Juan L, Menéndez S, Ocampo A, Mourelo J, Sáez JL (2012). Tuberculosis due to *Mycobacterium bovis*and *Mycobacterium caprae*in sheep. Vet J.

[15] Sharpe AE, Brady CP, Johnson AJ, Byrne W, Kenny K, Costello E (2010). Concurrent outbreak of tuberculosis and caseous lymphadenitis in a goat herd. Vet Rec.

[16] Cadmus SI, Adesokan HK, Jenkins AO, van Soolingen D (2009). *Mycobacterium bovis*and *M. tuberculosis*in goats, Nigeria. Emerg Infect Dis.

[17] Tschopp R, Bobosha K, Aseffa A, Schelling E, Habtamu M, Iwnetu R (2011). Bovine tuberculosis at a cattle-small ruminant-human interface in Meskan, Gurage region, Central Ethiopia. BMC Infect Dis.

[18] Islam KM, Rahman M, Islam MS, Adhikary GN, Rauf SMA (2014). Epidemiological studies of fascioliasis (*Fasciola gigantica*) in black Bengal goats. Eurasian J Vet Sci.

[19] Islam MH, Ripa RN (2015). Prevalence of fascioliasis in slaughtered goat in Bengal meat abattoir house and its economic impact on business. J Chem Biol Phys Sci.

[20] Afrin MK, Rahman AK, Dash K, Zaman S, Biswas PK, Sarker MS (2016). Study on diarrhea between PPR and fasciliasis in goat in Dinajpur Sadar veterinary hospital, Dinajpur. Eco-Friendly Agril J.

[21] Adedeji AJ, Dashe Y, Akanbi OB, Woma TY, Jambol AR, Adole JA (2019). Co‐infection of peste des petits ruminants and goatpox in a mixed flock of sheep and goats in Kanam, North Central Nigeria. Vet Med Sci.

[22] Karim A, Bhattacharjee U, Puro K, Shakuntala I, Sanjukta R, Das S (2016). Detection of peste des petits ruminants virus and goat pox virus from an outbreak in goats with high mortality in Meghalaya state, India. Vet World.

[23] Mesfine M, Nigatu S, Belayneh N, Jemberu WT (2019). Sero-epidemiology of foot and mouth disease in domestic ruminants in Amhara Region, Ethiopia. Front Vet Sci.

[24] Singh RK, Balamurugan V, Bhanuprakash V, Sen A, Saravanan P, Yadav M (2009). Possible control and eradication of peste des petits ruminants from India: technical aspects. Vet Ital.

[25] Barua N, Sutradhar BC, Chowdhury S, Sabuj AA, Torab A Sen A (2017). A case report on management of goat pox of a doe in Rangamati, Chittagong. J Biomed Multidiscipl Res.

[26] Alam J, Alam MS, Giasuddin M, Monoura P, Samad MA, Al Faruque MH (2016). Isolation, identification and molecular characterization of contagious ecthyma virus from goat and sheep. Bangladesh J livest Res.

[27] Babiuk S, Bowden TR, Boyle DB, Wallace DB, Kitching RP (2008). Capri poxviruses: an emerging worldwide threat to sheep, goats and cattle. Transb Emerg Dis.

[28] Rao TVS, Bandyopadhyay SK (2000). A comprehensive review of goat pox and sheep pox and their diagnosis. Anim Health Res Rev.

[29] Hamdi J, Bamouh Z, Jazouli M, Alhyane M, Safini N, Tadlaoui KO (2021). Experimental infection of indigenous North African goats with goatpox virus. Acta Vet Scand.

[30] Nadeem M, Curran P, Cooke R, Ryan C, Connolly K (2010). Orf: contagiou spustular dermatitis. Ir Med J.

[31] Nandi S, Ujjwal K, Sumit CD (2011). Current status of contagious ecthyma or orf diseases in goat and sheep-a global prescriptive. Small Rumin Res.

[32] APHIS Foot and mouth disease.

[33] Shanmugam Y, Muthukrishnan M, Singanallur NB, Villuppanoor SA (2015). Phylogenetic analysis of the leader proteinase (Lpro) region of Indian foot and mouth disease serotype O isolates. Vet Ital.

[34] Hoor EJM, Islam MS, Bari ASM, Khan MAHNA (2018). Pathology, serotyping and phylogeny of foot and mouth disease viruses circulated in the cattle of Sirajganj district, Bangladesh. Indian J Vet Pathol.

[35] Ranabijuli S, Mohapatra JK, Pandey LK, Rout M, Sanyal A, Dash BB (2010). Serological evidence of foot-and-mouth disease virus infection in randomly surveyed goat population of Orissa, India. Transb Emerg Dis.

[36] Birindwa BA, George GC, Ntagereka BP, Christopher O, Lilly BC (2017). Mixed infection of peste-des-petits ruminants and *Capripox*in goats in South Kivu, Democratic Republic of Congo. J Adv Vet Anim Res.

[37] Tebogo K, Andrew C, Choby C, Obed N, Beatus L, Emeli T (2019). Detection of peste des petits ruminants and concurrent secondary diseases in sheep and goats in Ngorongoro district, Tanzania. Comp Clin Path.

[38] Thombare NN, Sinha MK (2009). Economic implication of peste des petits ruminants (PPR) disease in sheep and goats: a sample analysis of District Pune, Maharastra. Agric Econ Res Rev.

[39] Settypalli TBK, Lamien CE, Spergser J, Lelenta M, Wade A, Gelaye E (2016). One-step multiplex RT-qPCR assay for the detection of peste des petits ruminants virus, *Capripox*virus, *Pasteurella multocida*and *Mycoplasma capricolum*subspecies (ssp.) capripneumoniae. PLoS One.

[40] Sultana S, Pervin M, Sultana N, Islam MR, Khan MAHNA (2022). Validation and standardization of designed *N*gene primer-based RT-PCR protocol for detecting peste des petits ruminants virus in goats. J Adv Biotechnol Exp Ther.

[41] Wilton S, Cousins D (1992). Detection and identification of multiple mycobacterial pathogens by DNA amplification in a single tube. PCR Methods Appl.

[42] Jiang XY, Wang CF, Zhang PJ, He ZY (2006). Cloning and expression of *Mycobacterium bovis s*ecreted protein MPB83 in *Escherichia coli*. college of biotechnology, Jilin Agricultural University, Changchun 130 118, China. J Biochem Mol Biol.

[43] Javed F, Mahbubur R, Samsul HP (2013). PCR based molecular detection of the *Gyr-B-2*gene from the *Klebsiella*sp. isolates from patients who were suffering with pneumonia and urinary tract infections (UTIs). J Clin Diagnostic Res.

[44] Ozmen O, Kale M, Haligur M, Yavru S (2009). Pathological, serological and virological findings in sheep infected simultaneously with bluetongue, peste-des-petits-ruminants and sheep pox viruses. Trop Anim Health Prod.

[45] Saravanan P, Balamurugan V, Sen A, Sarkar J, Sahay B, Rajak KK (2007). Mixed infection of peste des petites ruminants and orf on a goat farm in Shahjanpur, India. Vet Rec.

[46] Libeau G, Munir M (2015). Current advances in serological diagnosis of peste des petits ruminants virus. Peste des petits ruminants virus.

[47] Balamurugan V, Sen A, Saravanan P, Singh RP, Singh RK, Rasool TJ (2006). One-step multiplex RT-PCR assay for the detection of peste des petits ruminants virus in clinical samples. Vet Res Commun.

[48] Couacy-Hymann E, Roger F, Hurard C, Guillou J, Libeau G, Diallo A (2002). Rapid and sensitive detection of peste des petits ruminant’s virus by a polymerase chain reaction assay. J Virol Methods.

[49] Mahajan S, Agrawal R, Kumar M, Mohan A, Pande N (2014). Comparative evaluation of different *F*and *N*gene based reverse transcription polymerase chain reaction for molecular detection of peste des petits ruminants virus from clinical samples. Vet Arhiv.

[50] Pandey AK, Muglikar DM, Mhase PP, Pawade MM, Daphal SN, Pawar PD (2020). *F*gene and *N*gene based reverse transcription pcr for molecular characterization of peste des petits ruminants virus. Indian J Anim Res.

[51] Cadena AM, Ventura JD, Abbink P, Borducchi EN, Tuyishime H, Mercado NB (2021). Persistence of viral RNA in lymph nodes in ART-suppressed SIV/SHIV-infected Rhesus Macaques. Nat Commun.

[52] OIE (2009). Manual of diagnostic tests and vaccines for terrestrial animals.

[53] Berg S, Firdessa R, Habtamu M, Gadisa E, Mengistu A, Yamuah L (2009). The burden of mycobacterial disease in Ethiopian cattle: implications for public health. PLoS One.

[54] Kassa GM, Abebe F, Worku Y, Legesse M, Medhin G, Bjune G (2012). Tuberculosis in goats and sheep in afar pastoral region of Ethiopia and isolation of *Mycobacterium tuberculosis*from goat. Vet Med Int.

[55] Dauga C (2002). Evolution of the *gyrB*gene and the molecular phylogeny of *Enterobacteriaceae*: a model molecule for molecular systematic studies. Intl J of Syst Evol Microbiol.

[56] Khatun MS, Asaduzzaman M, Pallab MS, Chakrabartty P (2015). Risk factor analysis of fascioliasis in two geo-climatic regions of Bangladesh. Int J Sci Res.

[57] Islam KM, Islam MD, Rauf SMA, Khan A, Hossain MK, Sarkar S (2016). Effects of climatic factors on prevalence of developmental stages of *Fasciola gigantica*infection in *Lymnaea*snails (*Lymnaea auricularia*var. rufescens) in Bangladesh Islam. Arch Razi Inst.

